# Dosimetry of gamma chamber blood irradiator using PAGAT gel dosimeter and Monte Carlo simulations

**DOI:** 10.1120/jacmp.v15i1.3952

**Published:** 2014-01-04

**Authors:** Parvin Mohammadyari, Mehdi Zehtabian, Sedigheh Sina, Ali Reza Tavasoli, Reza Faghihi

**Affiliations:** ^1^ Nuclear Engineering Department Shiraz University Shiraz Iran; ^2^ Radiation Research Center Shiraz University Shiraz Iran; ^3^ Shiraz Blood Transfusion Organization Shiraz Iran

**Keywords:** blood irradiator, gel dosimetry, thermoluminescence dosimetry, Monte Carlo simulations

## Abstract

Currently, the use of blood irradiation for inactivating pathogenic microbes in infected blood products and preventing graft‐versus‐host disease (GVHD) in immune suppressed patients is greater than ever before. In these systems, dose distribution and uniformity are two important concepts that should be checked. In this study, dosimetry of the gamma chamber blood irradiator model Gammacell 3000 Elan was performed by several dosimeter methods including thermoluminescence dosimeters (TLD), PAGAT gel dosimetry, and Monte Carlo simulations using MCNP4C code. The gel dosimeter was put inside a glass phantom and the TL dosimeters were placed on its surface, and the phantom was then irradiated for 5 min and 27 sec. The dose values at each point inside the vials were obtained from the magnetic resonance imaging of the phantom. For Monte Carlo simulations, all components of the irradiator were simulated and the dose values in a fine cubical lattice were calculated using tally F6. This study shows that PAGAT gel dosimetry results are in close agreement with the results of TL dosimetry, Monte Carlo simulations, and the results given by the vendor, and the percentage difference between the different methods is less than 4% at different points inside the phantom. According to the results obtained in this study, PAGAT gel dosimetry is a reliable method for dosimetry of the blood irradiator. The major advantage of this kind of dosimetry is that it is capable of 3D dose calculation.

PACS number: 87.53.Bn

## I. INTRODUCTION

One of the most important problems that occur in blood transfusion is that infectious diseases transmit through contaminated blood products,^1^, ^2^, ^3^, ^4^ and this has become a major concern in current transfusion practices. Recently, blood and blood component irradiations have shown potential as a means to inactivate pathogenic microbes in infected blood products and to prevent graft‐versus‐host disease (GVHD) in immune compromised patients. These patients are susceptible to infection and, as a result are susceptible to infection by the abolishment of T‐lymphocytes, which are one of the most radiosensitive mammalian cells.^(5)^ Many investigators have reported required dose for inactivation of lymphocytes including Leitman,^6^, ^7^ who reported that gamma irradiation is the only proven method to inhibit transfusion associated GVHD and a 25 Gy dose is adequate for achieving that goal. Pelszynski et al.^8^, ^9^ reported that the minimum possible radiation dose of only 25 Gy is necessary for lymphocytes to be efficiently inactivated in red blood cell units. Also, Anderson et al.^10^, ^11^ mentioned that gamma irradiation is the only process that effectively prevents transfusion associated GVHD and suggested that blood components and products be irradiated to the lowest recommended dose (15–25 Gy) capable of preventing lymphocyte proliferation before blood transfusions. Nuclear radioactive sources like Cs‐137 are used to deliver doses to blood products, and errors in calculating the radioactive decay of the radiation source^(7)^ should be considered as a physical factor influencing the dose delivered to the blood product.

Based on this fact, the Food and Drug Administration (FDA) recommend a regular dose validation^(12)^ and verification of the recommended dose of 25 Gy to the midplane of the irradiated blood unit.^(13)^ So it is necessary for the dose rate and dose profile in the gamma chamber blood irradiator be measured. One of the basic design principles for all irradiators is to provide relatively uniform dose in the product.^(14)^ But variation of dose with depth and dose variation in the lateral direction contribute to the nonuniformity of the dose delivered to the products, so variation in dose in the irradiated product is unavoidable.^(14)^ Dosimetry is necessary for operational qualification of the irradiation facility.^(14)^ Different dosimeters are used to achieve this objective, for example, Fricke dosimeter and TL dosimeter, as well as GAFCHROMIC films. The first two dosimeters have limited resolutions, while GAFCHROMIC films have good resolution,^15^, ^16^, ^17^ but these dosimeters are 2D dosimeters. Gel dosimetry systems are true 3D dosimeters.^(18)^ No other conventional dosimeter is capable of fulfilling the requirements of a comprehensive 3D measurement of dose distribution in high‐dose gradients and in an irregular shape radiation field.^(19)^ PAGAT (PAG polymer gel and tetrakis (hydroxymethyl) phosphonium chloride (THPC) antioxidant) polymer gel dosimeter is considered to be one of the most promising dosimetry systems which has the potential to verify 3D dose distribution.^(20)^ According to the protocol of Shiraz Blood Transfusion Center, it is recommended that the dose profile be checked every six months; therefore, it is necessary to use an accurate dosimeter. In this study, dose profile of a blood irradiator was measured using PAGAT gel dosimeters and TLDs. The MCNP4C code in a real situation (dosimeters located beside blood bags) was used to calculate the dose and compare to the calculated dose with the gel dosimeter to determine whether it can be used to accurately measure the dose in a gamma cell blood irradiator.

## II. MATERIALS AND METHODS

### A. Blood irradiator

In this work a gamma cell blood irradiator (Gammacell 3000 Elan; Best Theratronic, Ottawa, Canada) in the Shiraz Blood Transfusion Center was used to irradiate cellular blood products to inactivate T lymphocytes in order to prevent graft‐versus‐host disease. The unit has four main components: radiation shield, sample chamber, radiation sources, and control system. The radiation source in the Gammacell irradiator is Cs‐137 in the form of powder embedded in two rod sources with total activity of 1356 Ci (50.2 TBq). Each Cs‐137 source is double encapsulated in stainless steel and permanently installed within the radiation shield. The sample chamber has a turntable and removable stainless steel beaker (12 cm diameter and 20 cm height) in which samples or blood products are placed.

### B. PAGAT gel dosimetery

#### B.1 PAGAT gel preparation

PAGAT gel was prepared inside a fume hood and under normal atmospheric conditions (temperature of approximately 20°C) using the PAGAT gel dosimetry recipe of Venning et al.^(21)^ After dissolving a certain amount of gelatin to the ultrapure deionized water for 12 min and heating to 48°C using an electric heater, the cross‐linking agent, bis, was added and stirred until completely dissolved. After that, acrylamide (AA) was added and stirred. Finally, the required amounts of the polymerization inhibitor hydroquinone (HQ) and the THPC antioxidant were combined with the polymer gel solution. The prepared gel was finally transferred into the vials and phantom and kept in a refrigerator at about 4°C. Table 1 shows the list of the elements and components used to manufacture this gel. The measured gel density was 1.02 g/cm^3^.

**Table 1:** Chemical components of PAGAT gel and concentration used

*Amount*	*Component*
5%	Gelatine (300 Bloom)
4.5%	N,N'‐Methylene‐bis‐Acrylamide (bis)
4.5%	Acrylamide (AA)
5 mM	Tetrakis‐Phosphonium Chloride (THPC)
0.1 mM	Hydroquinone (HQ)
86%	Deionized Water

#### B.2 PAGAT gel calibration

The response of the gel was calibrated using a Cs‐137 source calibrated by the secondary standard lab (SSDL) in Iran with the standard deviation of 3%. The dose rate at different distances from the Cs‐137 source was known. Fifteen cylindrical plastic vials of equal shapes and sizes (diameter: 1.5 cm, height: 10 cm) were filled with the gel and exposed to doses from 5 Gy to 32 Gy at a distance of 26.6 cm from the Cs‐137 source with the same medium dose rate of 8.7 Gy/hr to determine the linear range of its dose response. The vials were placed in the radiation field in order to ensure that all parts of them are irradiated with less than 2% dose variation across the gel dosimeters. An additional unirradiated vial of gel was used to determine the background level.

Once all the vials were irradiated, they were kept in the refrigerator for 4 hours before being sent to an MRI room for imaging. The characteristics of the MRI unit (Siemens MAGNETOM Avanto 1.5 T clinical MRI scanner; Siemens Medical Solution, Malvern, PA) used for imaging are listed in Table 2.

The $R^{2}(\text{transverse relaxation rate}\;(s^{-1}))$ of each vial was then obtained from the image, and the calibration curve of the PAGAT gel was prepared by drawing the $R^{2}$ value versus the radiation dose received by each vial. The $R^{2}$ value of each vial was calculated as the average of $R^{2}$ values in points inside a rectangle at the center of the vial, and the standard deviation of the $R^{2}$ values was also calculated from the $R^{2}$ values.

**Table 2:** The characteristics of MRI unit used for imaging

*Value*	*Parameter*
Multiple spine echo (32 echo)	Pulse sequence
256 mm	Field of view (FOV)
512×512	Matrix size
4 mm	Slice thickness (d)
4000 ms	Repetition time (TR)
22 ms	Echo time (TE)
4	Number of slices
32	Number of echoes
26 min	Total measurement time
0.5 mm	Resolution

### C. Dosimetry of the Gammacell blood irradiator using PAGAT gel

**Figure 1:**
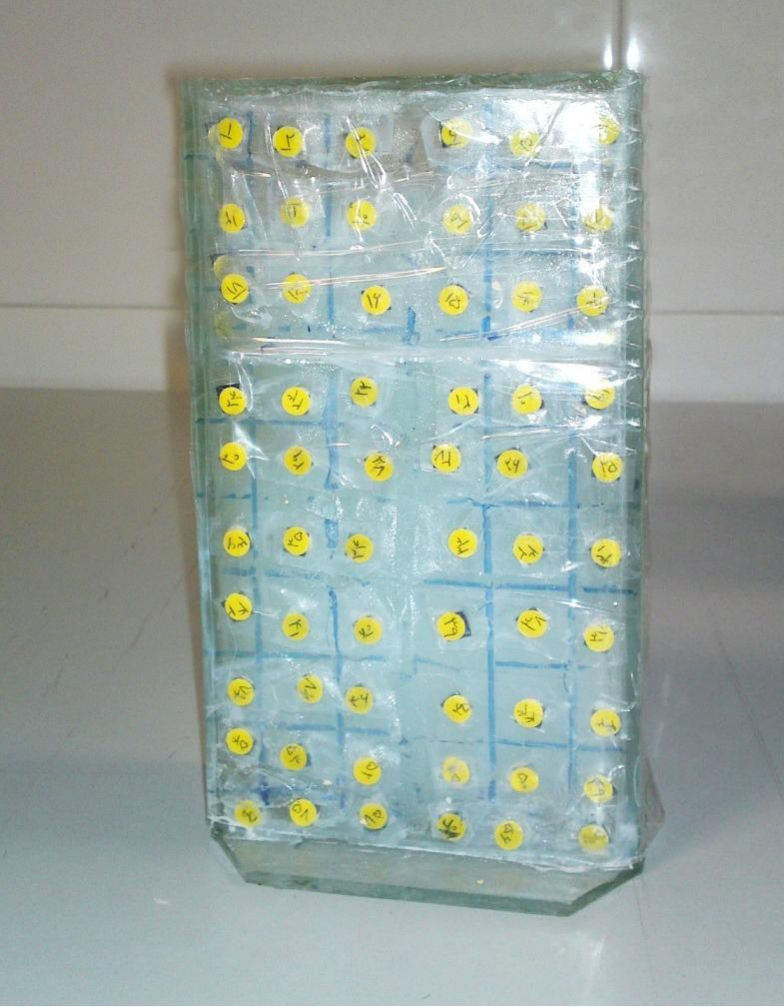
The phantom used for gel dosimetry with the TLD chips on the surface.

**Figure 2:**
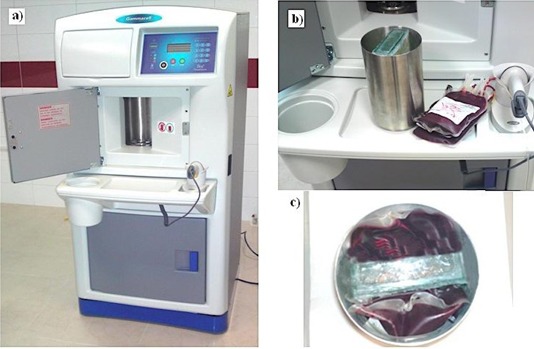
The blood irradiator (a), gel phantom (b), and gel phantom inside the blood container ready to be irradiated (c).

A glass phantom of dimension $10\times 4\times 18\;{\text{cm}}^{3}$ was built as the gel container. TLD chips were placed on the external surface of the gel container in order to compare the dose distribution measured by PAGAT gel with the results of TL dosimetry. The prepared PAGAT gel was then inserted inside the gel container and kept inside the refrigerator at about 4°C (see Fig. 1). The prepared gel containing phantom was irradiated by the Gammacell blood irradiator (model GC 3000 Elan, Company Best Theratonic, Canada) for 5 min and 27 sec. Figure 2(a) shows the blood irradiator and Fig. 2(b) shows our phantom inside the blood container ready to be irradiated.

Subsequently, the MRI image of the phantom was obtained, and the dose values at each point inside the phantom were assessed using the $R^{2}$ parameter of that point in the MR image and the PAGAT gel calibration curve.

### D. TL dosimeter

Sixty small TLD‐100 chips (LiF: Mg, Ti) with dimensions of 3.2 mm×3.2 mm×0.9 mm were used to determine dose profile at the center of the sample compartment. All TLD chips were annealed using a standard procedure (1 hr at 400°C, 24 hrs at 80°C),^(22)^ exposed to a known amount of dose and then read out by a Harshaw 4500 TLD reader (Thermo Fisher Scientific Inc., Waltham, MA) in order to estimate the efficiency calibration coefficients (ECC) values of each TLD chip. To determine the ECC values for each TLD chip, the readout of the TLD reader (in nC) for each of them was divided by the average readout of all chips. After determining the ECC values, the TLD chips were annealed and exposed to different doses to obtain the calibration curve, and afterward the TLD chips were annealed again to be ready for use in dosimetry. The TLDs were then placed along the surface of the phantom (Fig. 1) and transferred to the center of the beaker. After irradiation, the TLD chips were read out and the dose received by each TLD at each distance was determined from the calibration curve. It should be mentioned that in all TL dosimetry procedures, the time period between irradiation and readout of the TLDs was six days, which ensures that all the shallow traps were depleted.

### E. Monte Carlo simulations using MCNP4C code

**Figure 3:**
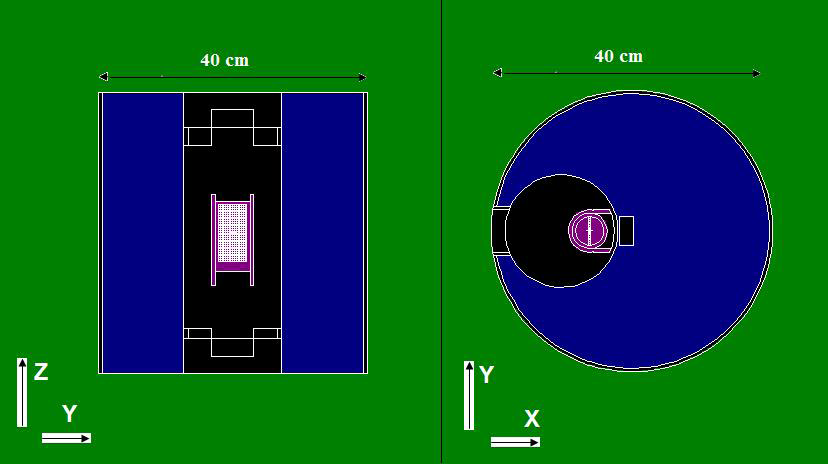
Two views of the simulation geometry used in MCNP4C simulations.

In this study, MCNP4C Monte Carlo code was used to simulate the blood irradiator components such as the source, shields, blood container, and the phantom that was used for dose calculation.^(20)^ MCNP codes have been used in many investigations, especially in radiotherapy dosimetry.^23^, ^24^, ^25^, ^26^ Two views of simulation geometry used for dose calculation are shown in Fig. 3. Tally type F6 was used in this study to score the absorbed dose at different distances of the source.^(25)^ A fine cubical lattice with the voxel size of 0.2 cm×0.2 cm×0.2 cm was defined at the center of the blood container in order to score the dose at each distance. As the source rotation in the MCNP4C cannot be simulated, several discrete sources on the periphery of the circle were defined and the isodose curves of each source were then added to those of others to assess the final isodose curve. The sources were defined as monoenergetic sources of energy 661.7 keV to simulate the Cs‐137 source. Finally, the results of the Monte Carlo simulations were compared with those obtained by other methods of dosimetry. The cutoff energy for both electrons and photons was taken to be 10 keV The number of $10^{9}$ particle histories was simulated for obtaining uncertainty of less than 1% at the tally cells.

As stated in previous section, TL dosimeters were placed on the surface of the phantom, while the dose estimations using Monte Carlo simulations and gel dosimetry were performed at the central slab of the phantom. Therefore, to obtain the correction factors for converting the surface dose measured by TLD to the depth dose, we have obtained the dose both at tally cells at the surface and also in the central slab of the phantom. For this purpose, the complete geometry of the blood irradiator was simulated, then a lattice with small cubical elements both on the phantom surface and inside the gel phantom was defined. For the correction factors converting the measured TLD dose to the phantom dose, we have divided the results of MC simulations at the surface in to the MC results of cubical elements inside the phantom. Using such factors for correction of beam attenuation in glass wall and gel phantom, we could convert the surface dose obtained by TLD to the dose at the central slab for comparison with gel and MCNP.

## III. RESULTS & DISCUSSION

**Figure 4:**
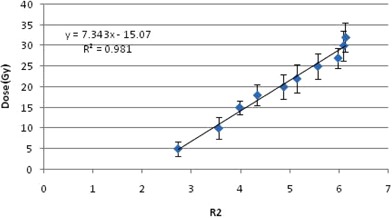
The calibration curve of PAGAT gel.

**Figure 5:**
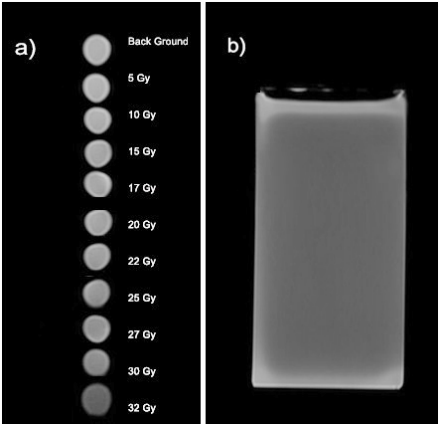
The MR image of (a) calibration vials at each dose, and (b) the phantom.

As mentioned in Materials and Methods section above, the response of PAGAT gel dosimeter taken at different dose, as well as the $R^{2}$ values, were obtained from the MR image. The calibration curve of the PAGAT gel was obtained. The calibration curve for the PAGAT gel has a linear response for doses from 5 to 35 Gy (Fig. 4). Figure 5 shows the MRI images of the calibration vials and the gel phantom. It should be noted that the SNR of the MR images was 100.

**Figure 6:**
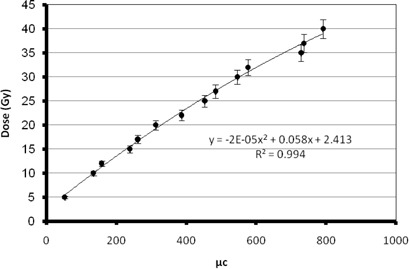
Calibration curve of TLD.

The dose values were obtained from the calibration curve of the gel at different points inside the phantom filled with the PAGAT gel. So these values were used for obtaining the isodose curve inside the phantom. The calibration curve of the TLD is also shown in Fig. 6. Tables 3 and 4 show the uncertainty analysis of thermoluminescence dosimetry and gel dosimetry. As can be seen from Table 3, the quadrature combination of statistical (Type A) and the systematic uncertainties (Type B) for TL dosimetry are 4.0% and 5.1%, respectively; for TL dosimetry, the total uncertainty is found to be 6.5%. The quadrature combination of Type A and Type B uncertainties for gel dosimetry are found to be 4% and 5.1%, with the total uncertainty of 6.8%.

**Figure 7:**
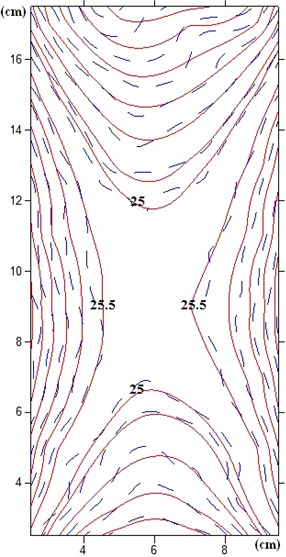
Comparison of isodose curves of PAGAT gel and the Monte Carlo simulations (dashed line=PAGAT gel;continuous line=MCNP4C simulations).

The dose at the fine lattice simulated inside the blood irradiator calculated by MCNP4C was also utilized for drawing the isodose curve. Figure 7 compares the results of PAGAT gel dosimetry with the results of Monte Carlo simulations. The results of the gel dosimetry are in close agreement with those of the MC simulations. According to the results of PAGAT gel dosimetry, the dose at all points inside the phantom varies between 22±0.2 to 28±0.3 Gy, with an average of about 25 Gy, while the dose values at the edges of the phantom along the y‐axis reach about 28 Gy, and the dose values at the phantom edges towards the z‐axis reach 22 Gy. This result shows that the maximum dose variations from 25 Gy (the desired dose for blood irradiation) is ± 12% at all points of the phantom. The two dimensional dose profile of the blood irradiator obtained in this study is in close agreement with the dose profile measured in previous investigations by GAFCHROMIC films.^(17)^

**Table 3:** Uncertainty determination in dose measurement using TLD (uncertainties within one standard deviation)

*Type B (%)*	*Type A (%)*	*Component of Uncertainty*
—	4.0	Repetitive TLD measurements
5. 0	—	TLD dose calibration (including uncertainty in calibration of Cs‐137 calibration source)
0	—	Correction for energy dependence of TLD
1.0	—	TLD positioning
5.1	4.0	Quadrature combination
6.5		Total uncertainty

**Table 4:** Uncertainty determination in dose measurement using gel dosimetry (uncertainties within one standard deviation)

*Type B (%)*	*Type A (%)*	*Component of Uncertainty*
—	3.0	Repetitive measurements
6. 0	—	Calibration of gel (including uncertainty in calibration of Cs‐137 calibration source)
0	—	Correction for energy dependence of gel dosimeter
1.0	—	Vial positioning
6.1	3.0	Quadrature combination
6.8		Total uncertainty

**Figure 8:**
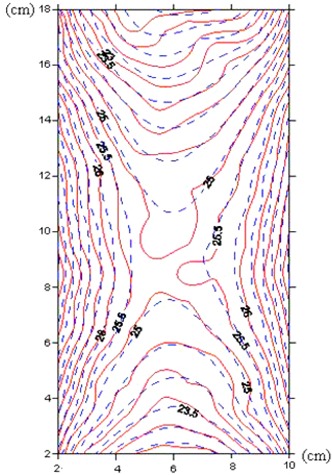
Comparison of isodose curves obtained by factory data and the Monte Carlo simulations (dashed line=MCNP4C simulation;continuous line=factory data).

The Gammacell 3000 Elan delivers a uniform dose of 25 Gy when targeted at the center of the canister. The isodose curve was obtained from the data given by the measurements of the factory. The isodose curve of the factory and the curve obtained by Monte Carlo simulation are compared in Fig. 8. As can be seen from the figure, the isodose curves obtained by the two methods are consistent, and the high dose uniformity at the center of the canister is confirmed.

**Figure 9:**
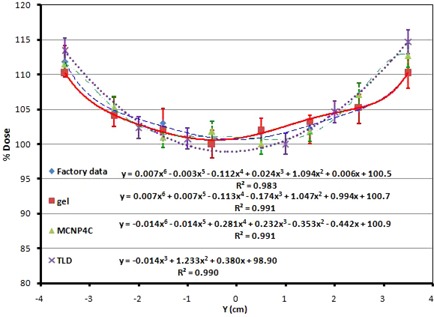
Dose profile along y‐axis at the central points, comparison of different methods (factory data, PAGAT gel dosimetry, Monte Carlo simulation, and TLD).

To obtain the dose profile along the y‐axis at the central points, the results of TL dosimetry, Monte Carlo simulations, PAGAT gel dosimetry, and the results given by the factory along the y‐axis were normalized to 100% for the minimum dose. The dose profile thus obtained is shown in Fig. 9. As expected, the dose along the y‐axis increases towards the edges of the phantom as we get near the source. According to the figure, the dose values obtained by different methods at the edges are 110% to 112% of the dose at the central point, which means 10% to 12% more than the dose at the central point. These results confirm the above‐mentioned maximum dose deviation of ± 12% at all points of the phantom. Such differences are mainly because of attenuation of the radiation with depth.

**Figure 10:**
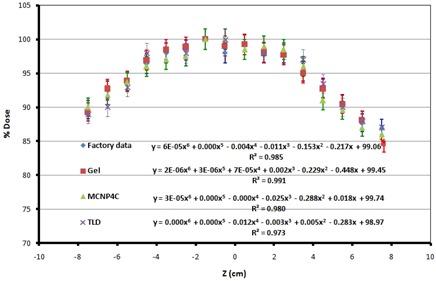
Dose profile along z‐axis, comparison of different methods (factory data, PAGAT gel dosimetry, Monte Carlo simulation, and TLD).

The dose profiles along the z‐axis at the central points for different methods were then obtained by normalizing the maximum dose (dose at central points) to 100% (Fig. 10). It is expected that the dose values along the z‐axis decrease when approaching the upper and lower edges of the phantom, as the distances between the source and the dose calculation points increase. According to the figure, the dose values obtained by different methods at the edges are 86% to 90% of the dose at the central point, which means 10% to 14% less than the dose at the central point. These results also confirm the maximum dose deviation of ± 12% at all points of the phantom. These differences are because of the dissimilarities of pencil source strength and nonuniform intersource spacing at cylindrical periphery.^(17)^ Figures 9 and 10 also compare the dose profiles of different methods that were compared with the results of the factory.

The percentage difference between the PAGAT gel dosimetry results and the Monte Carlo simulations ranges between 0.99% and 2.2%, with an average of 1.55%, while these values for TLD results and Monte Carlo simulations are 1.7% to 3.9%, with an average of 2.8%. The 6th order polynomials for the dose profiles in the y‐axis for each method, along with the $R^{2}$ values, are shown in Fig. 9. Therefore, the results of TL dosimetry and PAGAT gel dosimetry are in close agreement with those of Monte Carlo simulations. Figure 10 shows the 6th order polynomials and the $R^{2}$ values for the dose profiles in the z‐axis.

The percentage difference between the dose profile along the z‐axis obtained by PAGAT gel dosimetry and the results of Monte Carlo simulations range between 0% to 1.89%, with an average of 1.00%, and the comparison of the TLD results and Monte Carlo simulations shows that the percentage difference ranges between 0% and 2.8%, with the average of 1.1%.

**Figure 11:**
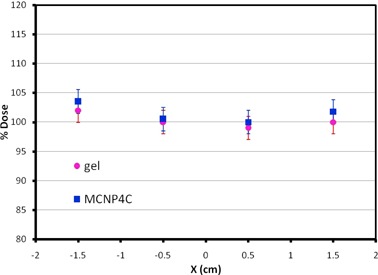
Dose profile along x‐axis, comparing PAGAT gel dosimetry and Monte Carlo simulation.

The dose profiles for x‐axis obtained by MCNP calculations and PAGAT gel are also compared in Fig. 11. The percentage difference ranges between 0.5% and 2%, with the average of 1.25%. As it is obvious, the results of Monte Carlo simulations are in good agreement with gel dosimetry results.

The dose profiles measured by gel dosimetry are in consistent with the previous investigations performed by GAFCHROMIC films.^(17)^ The most important advantage of gel dosimetry over the other dosimetry methods is the ability of three‐dimensional dose calculations, such that one can derive the dose profile at different slices of the phantoms using the MRI images.

## IV. CONCLUSIONS

The dosimetry of the Gammacell blood cell irradiator (model 3000Elan) was performed using PAGAT gel dosimetry, TL dosimetry, and MCNP4C Monte Carlo simulations.

In the case of gel dosimetry a phantom of dimensions $10\times 4\times 18\;{\text{cm}}^{3}$ was prepared to accommodate the gel dosimeter. Once the gel dosimeter was built using a standard procedure, the phantom was filled by the gel and irradiated in the blood irradiator (Cs‐137). The MR image of the irradiated gel was then obtained and the $R^{2}$ parameters of each point of the image were used to assess the dose at that point using the calibration curve. Sixty TLD chips were also put on the external surface of the phantom to estimate the dose on the phantom surface using the correction for attenuation and MC calculation. It should be mentioned that the ECC values of the TLD chips were determined three times, and the average of three measurements were used as the uncertainty of the calculated ECC factors, which varied between 0.2% and 0.8%, with the average of 0.7%.

The geometry of all the components of the blood irradiator was simulated by the MCNP4C Monte Carlo code, and the dose calculated in a fine cubical lattice inside the blood canister obtained by Tally F6 was used for drawing the isodose curves. The uncertainty of Monte Carlo calculations in all cells was less than 1%.

Verification of the recommended dose of 25 Gy to prevent the TA‐GVHD of the irradiated blood unit is critical, and the uniformity in dose delivery to the blood is a very important concept in blood irradiators. Therefore, it is necessary to measure the dose and dose profile in gamma chamber blood irradiator.

In the case of dose profile in y‐axis, the average percentage difference between the PAGAT gel dosimetry results and the Monte Carlo simulations is 1.55%, while this value is 2.9% for TLD results and Monte Carlo simulations. The average percentage difference in z‐axis between TLD, PAGAT gel dosimetry, and MCNP4C Monte Carlo simulations is about 1%. For the dose profiles in x‐axis, the average percentage difference between the MCNP calculations results and PAGAT gel is 1.25%.

The results of this study show that the absorbed doses determined from the gel and TLD dosimetry, MCNP4C calculations, and the factory data are in close agreement and therefore show that PAGAT gel can be used for accurate dosimetry of a blood irradiator. The advantage of the gel dosimeter is that it can provide three‐dimensional dose information.

## ACKNOWLEDGMENTS

The authors would like to thank the staff of the Radiation Research Center, Shiraz University, and Mr. Nahid and the staff of the MRI section of Moslemin Hospital, Shiraz.

## Supporting information

Supplementary Material

Supplementary Material
