# Supplementary figures and images for: Dosimetry of gamma chamber blood irradiator using PAGAT gel dosimeter and Monte Carlo simulations
Source: J Appl Clin Med Phys. 2014 Jan 4;15(1):317–30. doi: 10.1120/jacmp.v15i1.3952 (PMC5711240; doi:10.1120/jacmp.v15i1.3952)

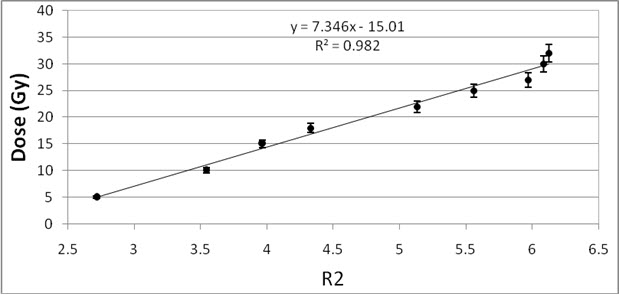

Supplement: Supplementary file 1 — Supplementary Material [file ACM2-15-317-s001.jpg]

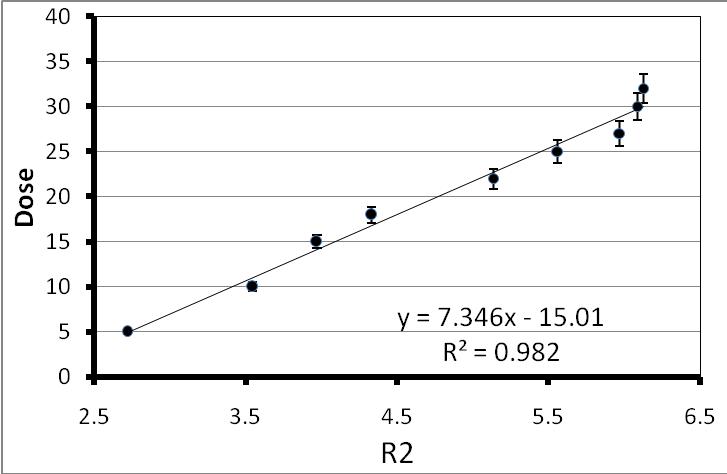

Supplement: Supplementary file 2 — Supplementary Material [file ACM2-15-317-s002.JPG]
